# Intradermal Administration of Fractional Doses of Inactivated Poliovirus Vaccine: A Dose-Sparing Option for Polio Immunization

**DOI:** 10.1093/infdis/jix038

**Published:** 2017-07-01

**Authors:** Hiromasa Okayasu, Carolyn Sein, Diana Chang Blanc, Alejandro Ramirez Gonzalez, Darin Zehrung, Courtney Jarrahian, Grace Macklin, Roland W. Sutter

**Affiliations:** 1 Research, Policy and Containment, Polio Eradication Department, and; 2 Expanded Programme on Immunization, Immunization, Vaccines and Biologicals Department, World Health Organization, Geneva, Switzerland; and; 3 PATH, Seattle, Washington

**Keywords:** fractional, polio, vaccine, intradermal vaccination, inactivated polio vaccine, vaccination.

## Abstract

A fractional dose of inactivated poliovirus vaccine (fIPV) administered by the intradermal route delivers one fifth of the full vaccine dose administered by the intramuscular route and offers a potential dose-sparing strategy to stretch the limited global IPV supply while further improving population immunity. Multiple studies have assessed immunogenicity of intradermal fIPV compared with the full intramuscular dose and demonstrated encouraging results. Novel intradermal devices, including intradermal adapters and disposable-syringe jet injectors, have also been developed and evaluated as alternatives to traditional Bacillus Calmette–Guérin needles and syringes for the administration of fIPV. Initial experience in India, Pakistan, and Sri Lanka suggests that it is operationally feasible to implement fIPV vaccination on a large scale. Given the available scientific data and operational feasibility shown in early-adopter countries, countries are encouraged to consider introducing a fIPV strategy into their routine immunization and supplementary immunization activities.

Providing vaccination through the intradermal route is an effective means of delivering vaccines due to the high concentration of immune cells in the epidermis (outermost layer of skin) [1]. Consequently, a lower intradermal dosage of some vaccines can potentially induce an equal or even higher immune response than a dose that is administered intramuscularly or subcutaneously, if administered correctly.

The concept of a reduced—or fractional—dose of inactivated poliovirus vaccine (fIPV) through intradermal administration has been evaluated in a number of clinical trials over the past 50 years. A study reported by Jonas Salk in 1953 using the first generation of IPV (before enhanced-potency IPV became available) concluded that polio vaccination given by both the intradermal route (using one sixth to one tenth of the standard dose) and intramuscular route demonstrated substantial immunogenicity [2]. Trials conducted between 1957 and 1979 (again using the nonenhanced IPV) [3], as well as pilot studies conducted with enhanced IPV in India [4–6] in the 1990s, found that, when provided as the primary series or as a booster dose, an intradermal fIPV dose of 0.1 mL (one fifth of the full 0.5-mL dose) induced a similar immune response as a full intramuscular dose. Despite these promising results, fIPV was not widely adopted except in Denmark, where it was the standard method of IPV vaccination in the mid-1950s [3].

In 2012, the World Health Assembly (WHA) endorsed the proposed Polio Endgame Strategy, which includes withdrawal of the Sabin-virus type 2 antigen—responsible for an estimated 95% of vaccine-derived cases of polio [7]—by replacing the trivalent oral poliovirus vaccine (tOPV) in the routine immunization schedule with a bivalent vaccine (bOPV) that lacks the type 2 Sabin virus. The Endgame Strategy also recommends the introduction of at least 1 dose of IPV to mitigate the risk of type 2 poliovirus resulting from the elimination of this component from the oral vaccine. Since the WHA resolution, all countries that were solely using OPV have either introduced IPV into their routine immunization schedule or decided to introduce IPV but have been unable to secure supply. The global demand for IPV has therefore substantially increased in just a few years.

At the same time, the WHA requested that the World Health Organization (WHO) work with partners and manufacturers to “enhance the affordability, effectiveness and accessibility [of IPV]” [8]. To fulfill this mandate, WHO has developed a multipronged strategy to reduce the cost of IPV, which includes fIPV as one of the most promising approaches [9].

The possibility of using fIPV has recently drawn more attention after the two global suppliers of WHO-prequalified IPV reported significant challenges in scaling up production to meet the new demand, resulting in a >50% reduction in their initial supply commitments made in 2014 (for the period 2014–2018). In many countries, these constraints have resulted in a delay in IPV introduction or stockouts, and they have affected the global IPV reserve established for outbreak response and supplementary immunization campaigns [10]. Therefore, the adoption of fIPV as an antigen-sparing technique could potentially be a key strategy for countries to stretch the limited supply of IPV while still ensuring high population immunity against polio.

Furthermore, even after polio eradication is achieved worldwide, countries will need to maintain population immunity against possible undetected polio transmission and accidental release of poliovirus from laboratories or vaccine production facilities [11]. Following eradication, countries will need to stop using OPV entirely due to the risk of vaccine-derived poliovirus emergence, possibly leading to endemic transmission. They will thus have to rely solely on IPV, further increasing the mid- to long-term demand for this vaccine. The fIPV strategy may provide an affordable solution to meet this increased demand for IPV in the post-OPV era.

In this context, we provide an overview of (1) the recent scientific literature on the use of fIPV for the primary series (either alone or in combination with OPV) and as a booster dose; (2) the development of new, improved devices for intradermal delivery of vaccines; and (3) the regulatory and operational aspects of using fIPV, including recent field experience.

## SUMMARY OF THE SCIENTIFIC EVIDENCE

### Studies of Fractional Inactivated Poliovirus Vaccine Used in the Primary Vaccination Series

#### Humoral Immune Responses

Humoral immunity (antibody response) protects an individual from paralytic disease. A series of clinical studies, many conducted within the past 5 years, have evaluated the humoral immunity of fIPV (using 0.1 mL, one fifth of the full 0.5-mL dose) administered intradermally through a variety of vaccine delivery devices (eg, Bacillus Calmette–Guérin [BCG] needles and syringes, hollow microneedles, needle-free jet injectors) compared with a full intramuscular dose when provided as primary vaccination in a 2- or 3-dose series using different vaccination schedules. These studies—conducted in Cuba [12, 13], Oman [14], Philippines [15], and Bangladesh [16]—evaluated immunogenicity by examining seroconversion rates and antibody levels following vaccination.

The studies found that cumulative seroconversion rates (a ≥4-fold increase over the expected decline in maternally derived antibody titers) for all polio serotypes following the complete vaccination series were comparable between the fIPV and intramuscular IPV groups when there was less interference with maternal antibody (that is, when the first dose was given at or after 2 months of age). On the other hand, the results were varied when the vaccination series started earlier (such as at 6 weeks of age). The Philippines study showed equivalent immunogenicity between fIPV and intramuscular IPV when each vaccine was administered at 6, 10, and 14 weeks of age [15]. However, studies in Cuba (using the same schedule of immunization at 6, 10, and 14 weeks) and in Bangladesh (using a 2-dose schedule at 6 and 14 weeks) showed slightly lower cumulative seroconversion rates in the fIPV group than in the intramuscular group [12, 16].

**Figure 1.**
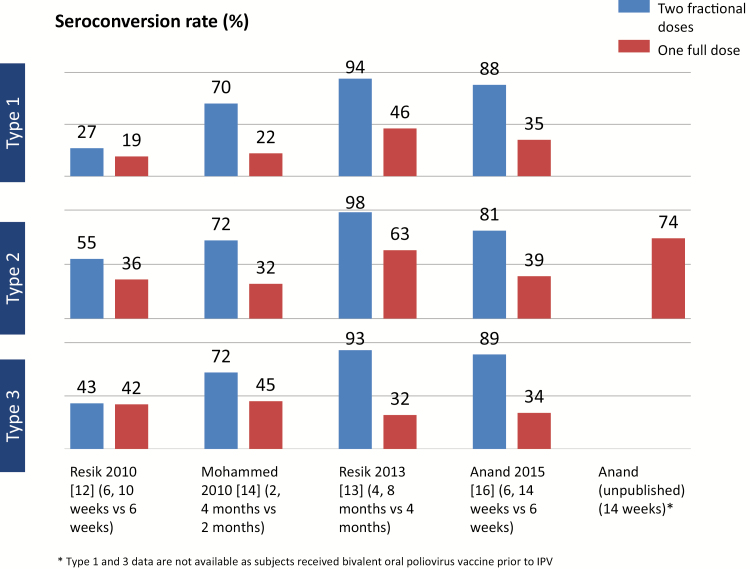
Comparison of seroconversion of 2 intradermal fractional inactivated poliovirus vaccine doses with 1 full intramuscular dose across 5 studies.

Because most OPV-using countries have added a single dose of IPV in their primary vaccination series, it is also useful to compare the immunogenicity of 2 fIPV doses with that of a single full intramuscular dose. In all studies, 2 fIPV doses (ie, total of 0.2 mL) resulted in substantially higher seroconversion rates for all poliovirus serotypes than a single intramuscular dose [12, 13, 14, 16, 17] (Figure 1).

Examining the median antibody levels by vaccination group in these studies, a single fIPV dose of 0.1 mL resulted in lower median neutralizing antibody titers (MATs) for any of the 3 poliovirus serotypes compared with a single full (0.5 mL) intramuscular dose. However, the MATs in the fIPV groups were all >1:8 dilution, indicating protection against paralytic disease. Thus, the difference in antibody levels between the fIPV and intramuscular groups was not considered clinically relevant [18]. In addition, median antibody levels following 2 fIPV doses were significantly higher in all studies than the titers following a single intramuscular dose for any of the serotypes.

After reviewing these data in October 2016, WHO’s Strategic Advisory Group of Experts on Immunization (SAGE) reiterated the recommendation it first made in April 2016 that countries should start preparing for a 2-dose fIPV schedule (eg, at 6 and 14 weeks of age), in lieu of a single intramuscular dose at 14 weeks [11]. Prior to the SAGE recommendation, 8 states in India and the country of Sri Lanka had already made this change to their immunization schedule. India will expand the use of fractional doses to an additional 8 states in August 2016 and to all 36 states in April 2017. In addition, Bangladesh has decided to introduce fIPV in their routine schedule in 2017 [19].

#### Mucosal Immune Responses

Although humoral immunity is important to prevent paralytic disease, mucosal immunity through the intestinal tract is essential to prevent excretion and transmission of the virus. Some of the studies mentioned above compared intestinal immunity induced by fractional versus full-dose IPV—with inconclusive results.

In the Oman study, infants received either a full intramuscular or intradermal fIPV at 2, 4, and 6 months of age, followed by a challenge dose of monovalent OPV type 1 (mOPV1) at 7 months [14]. Seven days after the challenge dose, the percentage of children shedding type 1 poliovirus was significantly higher in the fractional-dose group than in the full-dose group (74.8% vs 63.1%). In the Bangladesh study, a challenge dose of trivalent OPV was given to subjects at 18 weeks following vaccination with either fractional or full doses of IPV at 6 and 14 weeks [16]. Seven days after the challenge, viral excretion rates in the intramuscular arm and the fIPV arm were, respectively, 49.4% and 48.3% against type 1, 57.1% and 65.6% against type 2, and 32.1% and 42.4% against type 3. These differences were not statistically significant, suggesting that fractional intradermal doses were as good as full intramuscular doses of IPV in inducing intestinal immunity.

#### Duration of Seroprotection

A study in the Philippines compared seroprotection of fractional and full-dose IPV 12 to 15 months after infants received the primary series with 1 of these formulations at 6, 10, and 14 weeks [15]. Seroconversion rates for all poliovirus types were similar in the 2 groups: for example, 84% for type 2 poliovirus in the fIPV group versus 88% in the full intramuscular dose group. Geometric mean titers (GMTs) for type 2 poliovirus were 94.0 among infants in the fIPV group and 132.5 in the full-dose group. These results suggest that immunity induced by fIPV likely lasts as long as immunity induced by full-dose IPV.

### Using Fractional Inactivated Poliovirus Vaccine to Boost Immunity

Studies assessing the ability of fIPV to boost immunity among children who had previously received OPV have been conducted in India [20], Cuba [21], and Gambia [22]. These studies suggest that intradermal fIPV has less ability to boost antibody levels than the regular intramuscular dose, especially when the baseline titers are already high. However, recent studies among adults in Cuba [23] and among OPV-primed children aged 10 to 12 years in Sri Lanka (O. Mach, WHO, personal communication) found that an fIPV dose was as effective as a full intramuscular dose in boosting both humoral and mucosal immunity among OPV-immunized individuals. This suggests that fIPV through the intradermal route is as effective as IPV administered intramuscularly in boosting individuals with no or lower baseline titers.

Based on this new evidence, the WHO SAGE recommended in October 2016 that outbreak response campaigns (to boost population immunity) should use only fIPV [11].

### Intradermal Injection Devices

One of the challenges with the fIPV strategy is that intradermal injections are more complex to administer than intramuscular injections. Health workers must be well trained in the technique, and incorrect administration can lead to adverse events. However, new injection technologies have recently been developed and registered as alternatives to the conventional BCG needle and syringe to facilitate intradermal delivery of vaccines, including fIPV. These include several needle-free devices (eg, disposable-syringe jet injectors) and needle-based devices (eg, hollow microneedles and adapters that attach to the hub of the intradermal needle and syringe) (Table 1).

**Table 1.** Selected Devices for Intradermal Administration of Vaccines

Device (supplier)		Description	Registered for use	Doses per vial^a^	Availability of clinical data for IPV	Availability of clinical data for other vaccines
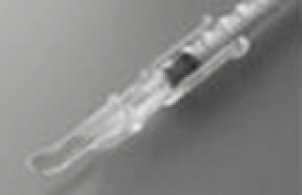	ID Adapter (West Pharmaceutical Services)	Plastic adapter that fits onto an autodisable intradermal needle and syringe that is provided with the device	Yes	5	Yes	No
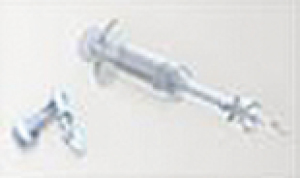	Star ID Syringe (Star)	Needle/syringe with a short minineedle and 90-degree injection angle, filled with an integrated plastic spike	No	5	Yes (prototype)	No
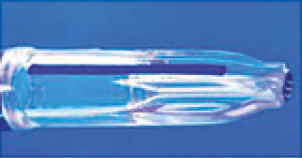	MicronJet 600 (NanoPass)	Hollow microneedle hub that can be attached to a luer syringe following filling with a separate needle	Yes	3	Yes	Yes
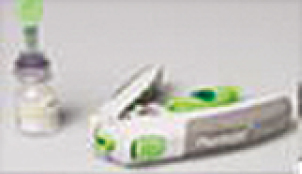	Tropis (PharmaJet)	Needle-free jet injector that uses a sterile single-dose syringe and pressurized liquid stream instead of needle	Yes	5	Yes	Yes
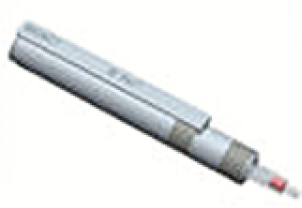	ID Pen (Bioject)	Needle-free jet injector developed as an alternative to Bioject’s gas-powered Biojector2000 device that is optimized for intradermal administration in low-resource settings (manually powered, intradermal only)	No	4	Yes	Yes

Abbreviations: ID, intradermal; IPV, inactivated poliovirus vaccine.

^a^Number of 0.1-mL doses obtained from each device from a model 0.5-mL vial [39].

Several of these devices have been tested in clinical studies comparing intradermal fIPV with intramuscular IPV, including the disposable-syringe jet injector Tropis (PharmaJet) in Gambia [22] and the MicronJet 600 (NanoPass) disposable hollow microneedle in Bangladesh [16] and in the United States [24]. These studies have demonstrated the safety and injection quality of these devices.

Other studies have assessed fIPV administration with novel intradermal devices alongside the conventional BCG needle and syringe, allowing a comparison of immunogenicity. In a recent study in Cuba [21], fIPV administered through 2 needle-free disposable-syringe jet injectors—Tropis and Biojector 2000 (Bioject)—achieved similar immune responses against all 3 poliovirus serotypes as those obtained with the BCG needle and syringe. However, a third jet injector—ID Pen (Bioject)—performed significantly worse, with seroconversion rates for type 2 poliovirus of 22.6% compared with 49%–54% for the BCG needle and 2 other injectors. Mild local reactions such as erythema, induration (hardening of the skin), and infiltration (swelling) were more frequent with all of the novel injectors than with the BCG needle and syringe. Healthcare workers preferred administering the vaccine with the disposable-syringe jet injectors over the traditional BCG needle and syringe because of the ease of administration with these devices.

In the Gambia study [22], OPV-immunized infants who had received a single dose of measles-rubella (MR) or yellow fever vaccine alone or in combination at 9–10 months of age (to study any interference between these vaccines and IPV) were given 1 dose of IPV 4–6 weeks later. The children were randomly assigned to receive the vaccine either via the intramuscular or intradermal route and by needle and syringe (both intramuscular and intradermal routes) or by disposable-syringe jet injectors (Stratis for intramuscular and Tropis for intradermal, both made by PharmaJet). Whereas both intradermal needle/syringe and intradermal jet injector groups had high seroprevalence rates (>93%) for all polio serotypes 28 days after the IPV dose, the median antibody levels in the intradermal jet injector (Tropis) group were identical for types 1 and 2 but lower for type 3. There were no differences in rates of local reactions (eg, erythema, infiltration, or tenderness) or systemic reactions (eg, fever, vomiting, excessive crying, poor appetite, diarrhea) between the groups vaccinated with a needle and syringe (either by intramuscular or intradermal administration) and those vaccinated using disposable-syringe jet injectors.

Another clinical study is currently taking place in Gambia to evaluate both the immunogenicity and the programmatic feasibility for use in immunization campaigns of a revised version of the Tropis device in comparison with other intradermal injection methods (needle and syringe, intradermal adapter).

Finally, a recent study in Pakistan compared the usability and immune response of fIPV using the intradermal adapter (West Pharmaceutical Services) and a novel intradermal syringe (Star Syringe) with the BCG needle and syringe [25]. It showed that the intradermal adapter induced a similar immune response (ie, seroconversion plus boosting) as the BCG needle and syringe and that vaccinators had a strong preference for intradermal adapters. The study also demonstrated that fIPV administration using either device was feasible and safe to use in both health center and campaign settings.

Intradermal delivery devices have also been positively evaluated in clinical studies of a number of other vaccines. These include influenza vaccination studies using the MicronJet 600 jet injector [26–29]; studies of various investigational nucleic acid vaccines (eg, dengue, herpes simplex virus, human immunodeficiency virus) delivered by the Biojector 2000 [30–32]; BCG vaccination with the Bioject ID Pen [33]; and studies of human papillomavirus (HPV), dengue, rabies, and various anticancer vaccines using the Tropis jet injector (C. Cappello, PharmaJet, personal communication). Intradermal devices have also been found to be preferred by parents and to cause less crying in infants compared with IPV delivery by intramuscular and intradermal injection with needle and syringe [12, 14, 25].

Upon review of available data, the Strategy Committee of the Global Polio Eradication Initiative (GPEI) endorsed in November 2015 the procurement of the intradermal adapter produced by West Pharmaceuticals and the Tropis (PharmaJet) disposable-syringe jet injector for the WHO IPV stockpile for polio outbreak response. These intradermal delivery devices would cost $0.50–$0.85 per injection, which is considerably more than conventional autodisable (AD) syringes ($0.04 per piece). However, a recent analysis conducted by PATH suggested that the dose sparing of fIPV can potentially offset the increased cost of these devices (Table 2). Further data are needed on operational costs, such as training health workers in intradermal administration, to expand on this initial cost analysis and draw firmer conclusions.

**Table 2.** Estimated Costs of Materials for Inactivated Polio Vaccination Per Immunized Child

Options	IPV	Device	Cost per child immunized (USD)	
			Delivery device	Total cost (vaccine + device)
Current strategy	Sanofi 10-dose vial (1 full dose)	IM AD needle and syringe	0.04	1.1
	BBio 5-dose vial (1 full dose)	IM AD needle and syringe	0.04	2.3
Proposed alternatives	BBio 5-dose vial (2 fractional doses)	ID AD needle and syringe	0.08	1.0
		ID adapter	1.1	2.0
		Jet injector	1.6	2.5
	BBio 1-dose vial (2 fractional doses)	ID AD needle and syringe	0.08	1.4
		ID adapter	1.1	2.4
		Jet injector	1.7	3.0

Key assumptions in cost estimates include the following:

Cost of vaccine per intramuscular dose: Sanofi 10-dose vial—$0.82; BBio 5-dose vial—$1.90; BBio 1-dose vial—$2.80.

Vaccine wastage rate: Single dose vial—5%; 5-dose vial—15%; >10 dose vial—20%.

Cost of devices: Intramuscular or intradermal autodiable needle and syringe—$0.04; Intradermal adapter with autodisable syringe—$0.55; Jet injector (including a needle-free syringe, filling adapter and device)—$0.75–$0.85 per injection.

Total uses per jet injector: approximately 1000, based on preliminary calculations of potential fractional inactivated poliovirus vaccine delivery volumes for 2 years during inactivated poliovirus vaccine supply shortage. Lifespan of device is ≥20 000 uses, but this could span many years in a routine immunization setting.

Abbreviations: AD, autodisable; BBio, Bilthoven Biologicals; ID, intradermal; IM, intramuscular; IPV, inactivated poliovirus vaccine.

## OPERATIONAL ASPECTS OF USING FRACTIONAL INACTIVATED POLIOVIRUS VACCINE

### Regulatory Considerations Concerning the Vaccine

Currently, the use of fIPV administered intradermally is considered an off-label use, and the IPV manufacturers do not intend to license their products for intradermal administration in the foreseeable future. However, it is not uncommon for public health authorities, such as the SAGE or national immunization technical advisory groups, to make recommendations that differ from indications on the vaccine product, especially in emergency situations, such as during yellow fever outbreaks [34]. Countries should therefore make decisions concerning the introduction of fIPV into their immunization schedule based on an independent scientific assessment of available clinical study data [11].

### Regulatory Considerations for Intradermal Injection Devices

The use of intradermal devices requires a separate regulatory clearance (eg, 510(k) in the United States and CE Mark in the European Union), depending on the country of use. The US Food and Drug Administration has recently added a new requirement that, for vaccines to be relabeled for use with disposable-syringe jet injectors, noninferiority clinical studies of the specific vaccine product using these devices must take place, in addition to the 510(k) clearance for the jet injector itself. The vaccine label can either indicate delivery by jet injection as a class of devices, enabling any 510(k)-cleared jet injector to be used, or specify a particular device [35]. The position of other national regulatory authorities on the requirement for label change specifically for jet injectors is less well defined. In 2013, WHO prequalified PharmaJet’s Stratis injector (used for intramuscular or subcutaneous injection) and requested that a jet injector “be used according to the label to deliver only those medications and vaccines that have been approved by the relevant Authorities” [36].

### Integrity of Vaccine Vial Stoppers

Using multidose vials involves piercing the vial stopper repeatedly with a BCG needle and syringe to draw out each dose. Fractional IPV significantly increases the number of piercings (ie, 5-fold), which could potentially compromise the integrity of the vial stopper. PATH recently conducted two types of tests of vial stoppers used for two WHO-prequalified IPVs following repeated piercing with a 27-gauge needle [37]. Self-sealing tests—in which the stoppers were pierced up to 100 times and the vials then submerged in methylene blue solution under a vacuum—did not demonstrate any closure failures with either stopper. Fragmentation tests—in which stoppers were pierced and the contents of the vial were then filtered and the visible particles counted—found that the frequency of fragments produced after up to 50 piercings met the target rate of <10% of punctures resulting in particle formation.

### Operational Feasibility of Using Fractional Inactivated Poliovirus Vaccine

Experiences in several states of India and in Sri Lanka have demonstrated that administering fIPV intradermally in a routine immunization setting can be done safely and effectively, provided there is a proper training program and supervision strategy.

The operational feasibility of administering fIPV doses during mass vaccination campaigns in response to outbreaks has also been demonstrated recently in India and Pakistan (both using a traditional BCG needle and syringe). An outbreak response campaign conducted in June 2016 in Hyderabad, India (in the state of Telangana, where routine immunization using fIPV was already taking place) demonstrated the feasibility of successfully planning and implementing a mass vaccination campaign using fIPV in a short period of time [38]. Within 14 days of the discovery of circulating vaccine-derived polio type 2 virus, >311 000 children aged 6 weeks to 3 years were vaccinated over a 6-day period using a fixed-site approach. Bleb formation (a small blister indicative of a correct intradermal injection) was observed in 93% of children, and no major safety concerns were found, nor was there leakage or visible damage to the vaccine vial cap or septum after repeated punctures. A postcampaign survey estimated a coverage rate for fIPV of 94% of target-age children. Another outbreak response campaign using fIPV was conducted among >200 000 children aged 4–23 months in Hyderabad, Pakistan, in October and November 2016.

To date, the only vaccine wastage data yet available for fIPV vaccination come from the mass vaccination campaigns conducted in India and the study conducted in Karachi, Pakistan, in 2016 [25]. In Hyderabad, India, where 10 full-dose IPV vials were used to give 50 doses of fIPV, a median of 48 doses (range = 41–50) were obtained per vial, for a median wastage rate of 4% [38]. In the Karachi study, where 5-dose IPV vials were used to obtain 25 doses of intradermal fIPV, the reported wastage rate was 10% using intradermal adapters and 6% using BCG needles and syringes [25].

## CONCLUSIONS

Considerable scientific evidence is available to support the use of intradermal fIPV for both routine and supplementary immunization activities. In addition, the development of a range of intradermal devices, such as needle-free jet injectors and needle adapters, show promise for furthering the use of fIPV by improving its usability and the ease of administration in the field. Given the severely limited global supply of IPV, countries are strongly encouraged to consider assessing the programmatic feasibility and trade-offs of introducing IPV using a 2-fractional-dose strategy through their national immunization technical advisory groups or other advisory bodies. This option would not only address the immediate IPV shortage but also serve as an affordable and immunogenic option for routine immunization after global polio eradication has been achieved. One possible strategy is to provide 2 fIPV doses beyond early infancy (eg, at the same time as diphtheria-tetanus-pertussis or measles) because this approach has been shown to result in seroconversion rates among children of >90% [13].

Moving forward, further assessments of the ongoing use of fIPV (eg, routine immunization in India and Sri Lanka, mass vaccination campaigns in Pakistan), as well as more pilot projects, would be helpful to facilitate country discussions and decision making to introduce fIPV. Additional research is also desirable to better understand the role of fIPV in inducing mucosal immunity and long-term immunity to provide further evidence to support the implementation of this strategy.

## Notes

***Financial support.*** This research did not receive any specific grant from funding agencies in the public, commercial, or not-for-profit sectors. The PATH authors’ contribution to this work was funded by a grant from the Bill & Melinda Gates Foundation.

***Supplement sponsorship.*** This work is part of a supplement coordinated by the Task Force for Global Health with funding provided by The Bill and Melinda Gates Foundation and the Centers for Disease Control and Prevention.

***Potential conflict of interest.*** All authors: No reported conflicts. All authors have submitted the ICMJE Form for Disclosure of Potential Conflicts of Interest. Conflicts that the editors consider relevant to the content of the manuscript have been disclosed.
